# Where economic parity meets ecology: Neither biodiversity nor ecosystem integrity values relate to wealth in the context of a medium-sized Finnish city

**DOI:** 10.1007/s13280-023-01927-z

**Published:** 2023-10-11

**Authors:** Sini Rautjärvi, Ian MacGregor-Fors

**Affiliations:** Ecosystems and Environment Research Programme, Faculty of Biological and Environmental Sciences, University of Helsinki, Niemenkatu 73, 15140 Lahti, Finland

**Keywords:** Biodiversity, Finland, Lahti, Urban ecology, Urban sociology, Wealth

## Abstract

Ecological conditions are heavily influenced by human–environment interactions, which is why understanding the relationships between people and nature is crucial. While earlier studies have indicated a pattern of positive correlations between economic wealth and biodiversity in urban areas, there are some examples that suggest that such associations are more intricate than initially presumed. In this study, we aimed to assess whether such a relation holds in Lahti, a medium-sized city in southern Finland, considering two biodiversity proxies (i.e., bird and woody plant species richness) and the Urban Ecosystem Integrity Index (UEII) of the city. Our results show no clear relationship between economic wealth (average annual income per statistical area) reported for 2019 and the two assessed biodiversity proxies and the UEII. These findings shed new light on the “luxury effect” in urban areas and reveal the nature of such relationship in highly green cities embedded in economic parity scenarios.

## Introduction

Cities are continually expanding and being established to accommodate a growing portion of the global human population (UN-Habitat 2022). However, our understanding of the ecological dynamics within these increasingly urbanized areas, where a rising number of people reside and depend of, remains limited (Grimm et al. 2008). Therefore, gaining a deeper understanding of the ecological mechanisms at play in cities becomes crucial (Lepczyk et al. 2017; Zari 2018). Given that cities function as socioecological systems, comprehending the interaction between human activities and the natural environment takes on added significance (Kowarik et al. 2020).

Urban ecology research has frequently emphasized the role of environmental factors as drivers of biodiversity within cities (Grimm et al. 2008). This emphasis has often been directed toward greenspaces, while the substantial coverage of the urban landscape by highly built structures and infrastructure, as well as the challenges faced by densely populated communities, have not received the same attention (Hope et al. 2003; Pickett et al. 2010). More recently, there has been a growing recognition of the significance of both the biological and social aspects of urban systems in comprehending their dynamics. This shift has led to an increased consideration of socioeconomics and culture, among other factors, when delving into the study of urban ecosystems (Grimm et al. 2008).

Woefully, the lack of access to greenery is often common across cities, highlighting the importance of socioeconomics when studying urban ecosystems (Strohbach et al. 2009; Nesbitt et al. 2019). A major challenge for future urban planning is preparing urban spaces for the growing population while simultaneously developing and maintaining cities as sustainable and livable places (Haase et al. 2017). Growing evidence from North American and European cities suggests an association between human wealth and urban biodiversity (e.g., Hope et al. 2003; Kinzig et al. 2005). According to Hope et al. (2003), there is a connection between the environmental quality and socioeconomic status in Arizona (USA), with plant diversity exhibiting a positive relationship with economic wealth. A similar pattern was recorded in Leipzig (Germany), where neighborhoods with higher income tended to have greater bird diversity (Strohbach et al. 2009). Accumulating evidence of these positive relationships between socioeconomics and biodiversity have led to coining of the concept known as “luxury effect” (Hope et al. 2003). Whenever this effect is present, the overarching goal is to counteract it and achieve an equitable distribution of biodiversity in cities (Leong et al. 2018). However, an increasing number of studies have found that the luxury effect does not appear to be generalizable, and can even vary in different contexts. For instance, Clarke et al. (2013) reported that median income in Los Angeles (California, USA) did not associate with tree diversity, but was related to legacy. Such “legacy effect” suggests that land management actions in the past continue to impact current urban vegetation cover and diversity (Clarke et al. 2013). Furthermore, in 2017, Blicharska et al. found no connection between aquatic insect diversity and socioeconomics in ponds from across of Stockholm (Sweden). Interestingly, Persson et al. (2018) showed that the link between biodiversity and socioeconomic conditions may be context-dependent. In urban areas of Stockholm, greater income related to lower urban greenery, while in suburban areas, higher income was linked to increased greenery (Persson et al. 2018).

The inconclusiveness of the luxury effect in urban areas might be related with the unique ways in which cities are established, managed, and planned, as well as the biodiversity proxy being assessed. However, it is evident that urban environmental inequality exists in cities worldwide (Pham et al. 2012). It is worth noting that the planning, establishment, and management of public and private urban greenery, which collectively form the greenspace network of cities, vary across regions, with economic forces often influencing these processes (Pham et al. 2012; Haase et al. 2017; Nesbitt et al. 2019). While public greenspaces in higher-income neighborhoods often correlate with biases in urban planning and urbanization across regions globally, wealthier areas can also enhance the ecological conditions of their immediate surroundings through investments on private greenery (e.g., gardens) (Leong et al. 2018; de Vries et al. 2020). Haase et al. (2017) clearly outlined a phenomenon known as eco-gentrification, in which housing prices and urban greenery are intertwined (de Vries et al. 2020). Increases in urban greenspace may lead to increases in housing prices, creating a cycle that makes it even more challenging for lower income urbanites to enjoy the numerous benefits of greener urban environment.

Among European cities, Finnish ones are known for their extensive green cover (Putkuri et al. 2013). According to Tiitu et al. (2017), greenspace cover, even in the downtowns of Finnish urban areas, accounts for 30–40% of the land. Following the global trend, the majority of Finns live in cities (Fina et al. 2021). Despite being a modern class society, Finland exhibits lesser gaps between social classes and lower economic inequality when compared to many other countries worldwide (although inequality is still increasing) (Melin 2020; Fina et al. 2021). Despite recent challenges, Finland continues to serve as a role model for the welfare state, known for its high levels of equality and social cohesion (Fina et al. 2021). It is noteworthy that an important trend in the Finnish population structure is aging—the proportion of pensioners is increasing, while the working-age population is decreasing (Kuntaliitto 2022). In 2021, the average age in Finland stood at 43.6 years, with important regional variations (Tilastokeskus 2022b).

Urban construction and building maintenance in Finland are subjected to strict legal regulations and oversight by authorities to ensure a healthy and safe environment that addresses the needs of everyone while being socially functional (Ministry of the Environment 2022). As well as building construction, greenspace establishment and management are also tightly regulated in Finland (Lahden kaupunki 2021b). The law requires public hearings of development plans (Slätmo et al. 2022), which places significant emphasis on incorporating public opinions and participation into urban planning, including environmental considerations (Lawrence 2022).

In this study, we examined the relationship between average annual income per statistical area (referred to as income hereafter) and two biodiversity proxies—bird and woody plant species richness—as well as the recently proposed Urban Ecosystem Integrity Index (UEII) in the city of Lahti (southern Finland). Focusing on these three explanatory variables enabled us to form a comprehensive understanding of the environmental conditions throughout Lahti. Given the significant green cover of Lahti (~ 51%, MacGregor-Fors et al. 2022) and the comparatively low economic inequity of Finnish cities in comparison to other regions, we did not expect correlations between either the two biodiversity proxies or the UEII and economic wealth.

## Materials and methods

### Study area and survey sites

We conducted this study in Lahti, a city and municipality located in the Päijät-Häme region in southern Finland (~ 120,000 inhabitants, 60° 59′ 01″ N, 25° 39′ 23″ E; average 100 m asl). Ranked as the sixth most populated urban center in Finland, the urban continuum of Lahti covers an area of 54 km^2^ (MacGregor-Fors et al. 2021). The city’s location at the intersection of railway, water, and land transportation, coupled with the establishment of the Riihimäki (Finland)–St. Petersburg (Russia) railway in 1870 and the opening of the Vääksy canal in 1871, facilitated its growth and development (Lahden seutu guide 2017). This development continued post-wars, with Lahti becoming a hub of industrialization in Finland during the 1960s and 1970s (Landström 2017).

Today, Lahti is recognized for its commitment to environmental sustainability and its engagement in active research that intertwines the ecological, social, and physical dimensions of the city (MacGregor-Fors et al. 2021). In 2021, Lahti achieved the distinction of being the first Finnish city to be awarded the title of European Green Capital, with a goal of achieving carbon neutrality by 2025 (Lahden kaupunki 2021a; European Commission 2022). More recently, Lahti received the Covenant City in the Spotlight Awards 2022 (medium-sized signatory category) for its notable progress in climate mitigation (Covenant of Mayors 2022). Economically, the gross domestic product per capita in the Päijät-Häme region, of which Lahti is the capital, was ranked lowest in Finland, which stands in stark contrast to the national average per capita gross domestic product (Tilastokeskus 2021). To provide numerical perspective on Lahti’s economic standing within Finland, the average yearly income in Lahti by statistical area for 2019 was of €27,830 (TILDA 2022; Table 1), while in the same year was €40,943 in Helsinki, €30,464 in Tampere, and €28,338 in Turku (Tilastokeskus 2022a). According to Kuntaliitto (2022), the proportion of individuals over the age of 65 in Lahti has increased from 15.3 to 25.2% over the past decade (2001–2021), slightly surpassing the national average (23.1%, Kuntaliitto 2022).

**Table 1:** Average (± SD) income, UEII, bird, and woody plant species richness across Lahti’s 2019 income quartiles by statistical area

	Quartile			
	1	2	3	4
Income (mean)	€21,730.33	€26,160.98	€29,222.28	€34,246.53
Income (SD)	€2437.46	€1470.63	€747.01	€2290.20
UEII (mean)	0.21	0.10	0.18	0.11
UEII (SD)	0.36	0.39	0.33	0.33
Bird (mean)	6.63	5.90	6.26	6.02
Bird (SD)	2.17	2.12	1.89	2.31
Plant (mean)	7.35	7.24	7.91	7.80
Plant (SD)	4.09	4.64	4.08	3.96

### Biodiversity proxy data

**Fig. 1:**
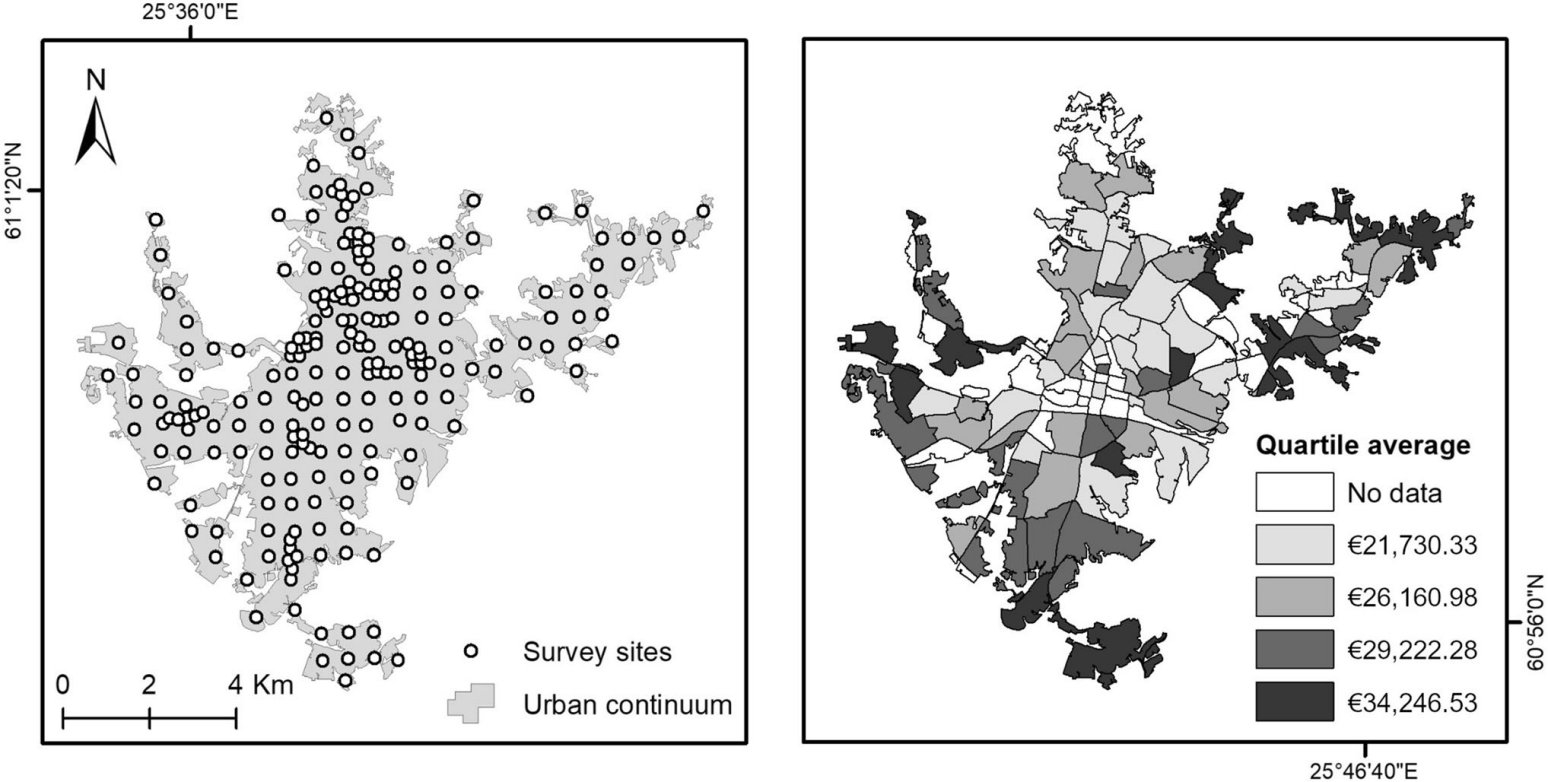
Location of the biodiversity proxy and UEII survey sites (left). Average annual income per statistical area within the urban continuum of Lahti (right)

A citywide survey considering the urban continuum of Lahti was followed to document bird and woody plant species richness during the summer of 2021. The polygon delineating this continuum was generated using satellite images and a set of criteria considering building aggregation and road communication with the aim of encompassing all the continuous built-up infrastructure of the city (see MacGregor-Fors 2010; Lemoine-Rodríguez et al. 2019). A 600 × 600 m grid was overlaid onto the city’s polygon, where survey locations were established in the centroid of each cell, resulting in 157 survey sites. An additional set of 60 survey sites was manually placed within the largest greenspaces of the city, with a minimum distance of 250 m between each other to ensure spatial independence of bird data. Thus, the total number of survey sites for this study was 217 (Fig. 1). Bird species richness was assessed by performing one 10-min point-count (50 m radius) at each survey site. For practical reasons, woody plant richness was surveyed in 25 m radius plots.

### Urban Ecosystem Integrity Index

The UEII is a novel user-friendly tool designed for evaluating ecosystem integrity within cities. Ecosystem integrity was originally defined by Karr and Dudley (1981) as “the capability of supporting and maintaining a balanced, integrated, adaptive community of organisms having a species composition, diversity, and functional organization comparable to that of natural habitat of the region.” The UEII contrasts the biological and the physical components of sites within cities to reference systems that resemble the conditions that existed in the region of a given city before urbanization (MacGregor-Fors et al. 2022).

As explained in detail by MacGregor-Fors et al. (2022), we generated interpolated raster layers for both bird and woody plant species richness using the inverse distance weighting (IDW) interpolation technique. The physical aspect of the UEII was based on built cover (assessed through an atmospherically corrected Sentinel 2 L2A image with a spatial resolution of 10 m) and land surface temperature (LST, °C; retrieved from a 100 m Landsat 8 OLI/TIR band resampled to 30 m). For additional details on the procedures and calculations related to the UEII, see MacGregor-Fors et al. (2022).

### Economic wealth data

The economic wealth data for 2019, pertaining the population aged 15 years and over, were retrieved from the publicly available City of Lahti statistical database TILDA (2022). Lahti is divided into 169 statistical areas and 15 income classes (taxable income). Using these data, average income for each statistical area was calculated. Thus, the dataset used in this study includes a total of 89,121 Lahtians, excluding those who live outside the city’s urban continuum. Notably, not all statistical areas contained the same amount of survey points for the biodiversity proxy data and UEII; some even had no survey sites at all (1.65 ± 1.86; maximum = 8). Additionally, three of the surveyed sites were not contained in any of the assessed statistical areas of Lahti and were thus not included in the analysis.

### Data analysis

We ran a linear model in R (R Core Team 2020) to assess the relationships between Lahti’s income and the three independent variables: bird species richness, woody plant richness, and the city’s UEII. We log transformed the woody plant species richness data due to its non-normal distribution. Considering the correlation between the three independent variables in our study (birds–plants: *r* = 0.29, *P* < 0.001; birds–UEII: *r* = 0.59, *P* < 0.001; plants–UEII: *r* = 0.73, *P* < 0.001), we examined the potential for variance inflation factors (VIF) in our model. While some consider VIF ≥ 10 indicative of collinearity (e.g., Hocking 2003), others propose lower thresholds, such as ≥ 5 (as noted by Gareth et al. 2023). Therefore, considering the nature of our data and our concern for potential collinearity, we set a VIF threshold of 5 for this study. Finally, we calculated the coefficient of determination (*r*^2^) of the model to quantify the proportion of the variance of the dependent variable explained by the independent ones.

## Results

**Fig. 2:**
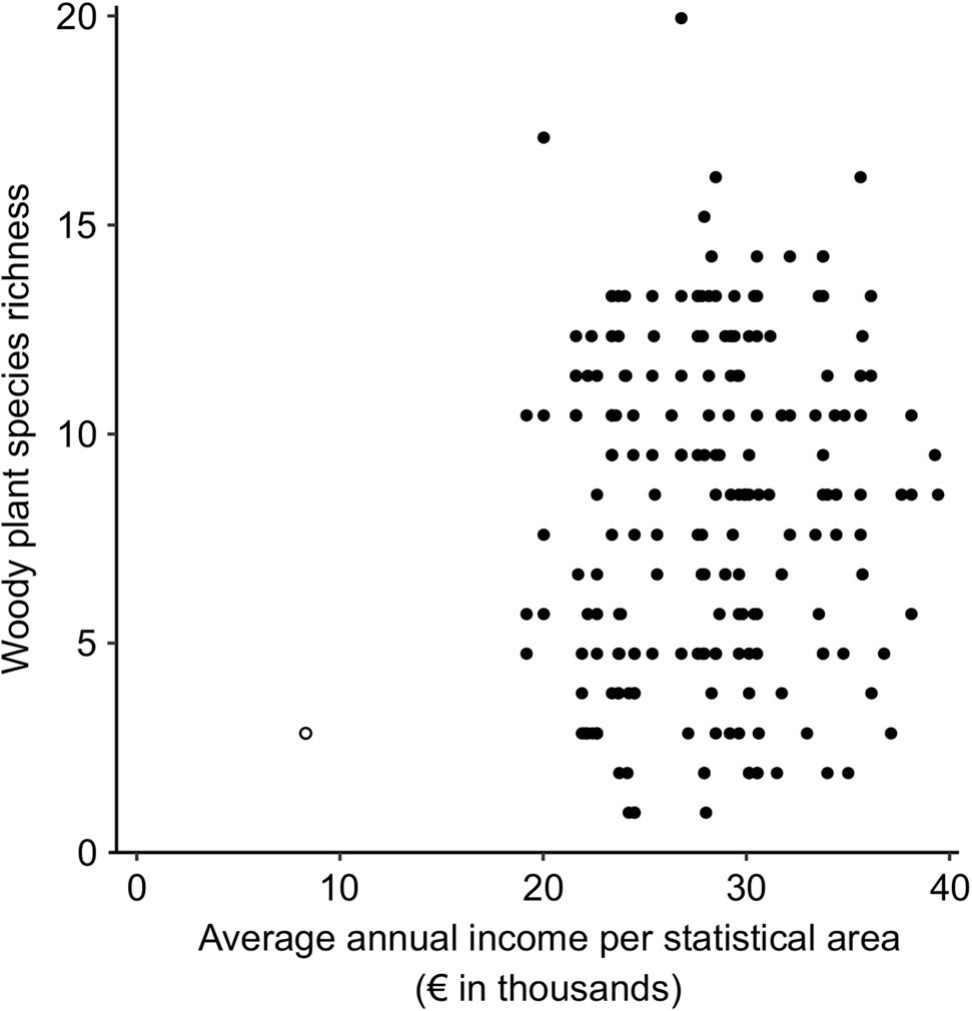
Relationship between average annual income per statistical area and the plant species richness recorded across the citywide survey of Lahti. The white circle depicts the data for Mukkulan Kartano

The linear model, which exhibited no indications of collinearity concerns (all VIF values ≤ 3.01), showed a positive relationship between woody plant species richness and income in Lahti (Table 2); however, the model’s explanatory power was limited (*r*^2^ = 0.035; Fig. 2). Upon scrutinizing the association between woody plant richness and income in Lahti, we recognized the potential influence of a single data point. This point, Mukkulan Kartano, corresponds to our dataset’s lowest-income statistical area and had only two woody plant species in the surveyed area (depicted with a white circle in Fig. 2). Upon excluding the data from Mukkulan Kartano, the new model continued to show a positive significant relationship between woody plant richness and income in Lahti. Notably, in the second model, the *P* value (*P* = 0.022) increased compared to the one including Mukkulan Kartano, while the explanatory power decreased further (*r*^2^ = 0.027).

**Table 2:** Multivariate linear model assessing relationships between Lahti’s average annual income per statistical area and bird species richness, woody plant richness, and the city’s UEII

Variable	*F*	*P*
Bird richness	0.119	0.730
Woody plant richness	7.073	0.008
UEII	0.180	0.671

## Discussion

The aim of this study was to assess the relationship between income and two biodiversity proxies, along with the UEII of the city of Lahti. The outcome of the linear model only showed a positive and significant relationship between woody plants and income in Lahti. Yet, the model’s explanatory power was so minimal that such a relationship may be considered negligible (Fig. 2). Given that *P* values tend to decrease with larger sample sizes (Lin et al. 2013), the interpretation of significant relationships in such scenarios demands caution, emphasizing not only *P* values but also the explanatory power of models (as highlighted by Altman and Krzywinski 2017).

Previous research on the relationship between economic wealth and urban biodiversity remains inconclusive. While an increasing number of studies have shown positive relationships (e.g., Kinzig et al. 2005; Strohbach et al. 2009; Pham et al. 2012; Hope et al. 2003), our findings are in agreement with the handful of evidence finding no direct relationship between income and urban biodiversity (e.g., Clarke et al. 2013; Blicharska et al. 2017). However, it is important to note that in this study we did not assess potential spatial variations, which a recent study has suggested could explain the relationships between economic wealth and urban biodiversity (Persson et al. 2018).

One potential factor contributing not to find a relationship between income and urban biodiversity in Lahti could be Finland’s narrow social class differences and low economic inequality compared to many other nations (Melin 2020; Fina et al. 2021). Furthermore, like most Finnish cities, Lahti is characterized by abundant greenery (Putkuri et al. 2013; Tiitu et al. 2017; MacGregor-Fors et al. 2021). These factors seem to play a major role on driving biodiversity and the UEII of Lahti, overshadowing the potential importance of socioeconomic variables.

The absence of a correlation between income and biodiversity in Lahti provides support for the evidence suggesting that the luxury effect only occurs in specific scenarios, further emphasizing a more intricate and context-dependent relationship. Thus, our findings contribute to the growing body of literature suggesting that the drivers of biodiversity in urban areas are manageable, with factors such as built surface and greenspace availability playing crucial roles (Rega-Brodsky and MacGregor-Fors 2023). Additionally, other socioeconomic factors may play significant roles in urban biodiversity, especially when their distribution across cities is uneven (Andrade et al. 2023; Kendal et al. 2023). In cities where notable disparities of socioeconomic factors exist, considering their impact becomes essential when analyzing urban biodiversity patterns (Kinzig et al. 2005).

Given that our study focused on a single medium-sized city situated in southern Finland, our findings need to be contextualized accordingly. Besides the socioeconomics of Finland, and specifically Lahti, it is important to consider factors such as its aging population and the impact of a significant number of pension recipients when analyzing annual income. According to the Finnish Centre for Pensions (Eläketurvakeskus 2022), the average monthly pension in 2021 amounted to €1784, with over half of pensioners receiving less than €1500 per month. This context gains significant relevance when analyzing the distribution of annual income in Lahti, given the city’s substantial population of pensioners and the comparatively lower pension amounts in contrast to regular wages.

Drawing from our own findings and the existing literature, we foresee future research efforts to adopt a multi-city systematic approach. This can be achieved through controlled surveys conducted across a collection of cities with diverse circumstances and characteristics. This approach could unravel not only citywide patterns, but could also capture regional variations. To enhance our comprehension of the phenomenon, future research would benefit of incorporating a broader set of explanatory variables, including the health, social, cultural, economic, built, and environmental dimensions of cities.

## Conclusion

As urbanization continues to rise, comprehending the connections between people and nature within cities becomes crucial. Through examining the relationships between income and biodiversity indicators in a mid-sized Finnish city, this study revealed results that challenge much of the existing research. Neither biodiversity nor ecosystem integrity values exhibited meaningful correlations with income in Lahti. This study’s findings, together with recent research indicating no apparent links between income and urban biodiversity (or even context-dependent situations within the same city; e.g., Persson et al. 2018), offer valuable insights into future urban management and city planning strategies.
